# Maturation of Neural Cells Leads to Enhanced Axon-Extracellular Matrix Adhesion and Altered Injury Response

**DOI:** 10.3389/fbioe.2020.621777

**Published:** 2021-01-06

**Authors:** Xueying Shao, Maja Højvang Sørensen, Chao Fang, Raymond Chuen Chung Chang, Zhiqin Chu, Yuan Lin

**Affiliations:** ^1^Department of Mechanical Engineering, The University of Hong Kong, Hong Kong, China; ^2^HKU-Shenzhen Institute of Research and Innovation, Shenzhen, China; ^3^Department of Electrical and Electronic Engineering, Joint Appointment With School of Biomedical Sciences, The University of Hong Kong, Hong Kong, China; ^4^Laboratory of Neurodegenerative Diseases, Li Ka Shing (LKS) Faculty of Medicine, School of Biomedical Sciences, The University of Hong Kong, Hong Kong, China

**Keywords:** neuron adhesion, axon retraction, traumatic injury, neuron mechanics, cell adhesion

## Abstract

Although it is known that stronger cell-extracellular matrix interactions will be developed as neurons mature, how such change influences their response against traumatic injury remains largely unknown. In this report, by transecting axons with a sharp atomic force microscope tip, we showed that the injury-induced retracting motion of axon can be temporarily arrested by tight NCAM (neural cell adhesion molecule) mediated adhesion patches, leading to a retraction curve decorated with sudden bursts. Interestingly, although the size of adhesion clusters (~0.5–1 μm) was found to be more or less the same in mature and immature neurons (after 7- and 3-days of culturing, respectively), the areal density of such clusters is three times higher in mature axons resulting in a much reduced retraction in response to injury. A physical model was also adopted to explain the observed retraction trajectories which suggested that apparent adhesion energy between axon and the substrate increases from ~0.12 to 0.39 mJ/m^2^ as neural cell matures, in good agreement with our experiments.

## Introduction

Strong attachment to the extracellular matrix (ECM) is critical for neural cells to execute biological duties such as information transmission (Fields and Stevens-Graham, 2002; Togashi et al., 2009), memory consolidation (Sandi, 2004; Washbourne et al., 2004), and nerve regeneration (Yu et al., 2008; Togashi et al., 2009; Eva and Fawcett, 2014; Nieuwenhuis et al., 2018). Microscopically, the binding of transmembrane proteins like neural cell adhesion molecule (NCAM) and N-cadherin to their counterparts from the ECM or another cell is believed to bring two surfaces together (Yu et al., 2008; Liu et al., 2020). Interestingly, as a neuron matures, its axon undergoes significant cytoskeletal changes which also lead to an altered adhesion capability to the outside (Doherty et al., 1992; Kamiguchi, 2007). For example, periodic membrane skeleton (Zhong et al., 2014) will be developed in mature axons while such organized structure is often missing in immature ones. In addition, adhesion proteins such as integrin were found to be widely distributed in the membrane of immature neural cells whereas their expression become relative low after neuron maturation (Eva and Fawcett, 2014; Nieuwenhuis et al., 2018).

It is conceivable that these maturation-induced changes could profoundly affect neurons in performing biological functions or responding to stimuli from outside. Indeed, it has been reported that a growth cone can often be reformed at the transected end of an immature axon whereas no such phenomenon was observed on mature neural cells (Nieuwenhuis et al., 2018; Shao et al., 2019; Wang et al., 2020). Our previous studies also revealed that physical injury of axons can trigger their retraction, a motion that is believed to be driven by internal tension inside axon and resisted by cell-substrate adhesion (Shao et al., 2019). However, whether and how (in indeed) mature and immature neurons respond differently to injury, as well as the reasons behind, remain unclear. This issue is of great importance clinically because neurogenesis from neural stem cells (Christian et al., 2014) has been increasingly realized as a promising treatment strategy for traumatic brain injury (TBI) to compensate for the loss of neurons and disruption of the neural network (formed by interconnecting axons) in patients suffered during, for example, falls or car crashes (Scheid et al., 2006; Johnson et al., 2013; Sharp et al., 2014). Interestingly, recent evidence showed that TBI can actually promote neurogenesis of newly developed neurons but not on well-developed ones (Braun et al., 2002), indicating a precise knowledge on the injury response of mature and immature neural cells could be one of the keys for treating this disease in the future.

Here, we reported a combined experimental and modeling investigation to address this question. Specifically, the spatial distributions of NCAM-mediated adhesion clusters formed between nascent and mature axons and the substrate were quantified by TIRFM (total internal reflection fluorescence microscopy) imaging. In addition, the retraction response of mature and immature axons, induced by axotomy with a sharp AFM tip, was closely monitored and compared. Finally, a physical model was adopted to explain the observed retraction trajectories as well as connect them with the observed adhesion patterns at the axon-substrate interface.

## Experiment Methods

### Cell Preparation

Primary cortical neurons were obtained by dissecting the brain of embryonic 17-day-old Sprague-Dawley Rats, provided by Laboratory of Neurodegenerative Diseases (The University of Hong Kong). Five milligram of poly-L-lysine (PLL) powder was dissolved in sterile PBS and 2 mL of such PLL solution was added on a glass bottom culture dish (MatTek Corporation, 35 mm Dish, 10 mm Glass Diameter) overnight. The PLL solution was then removed and the dish was rinsed with autoclaved milli-Q water once. Neurons were then seeded on the resulting PLL-coated dish, at 37°C and with 5% CO_2_ supply, until a density of 3.5 × 10^5^ cells/dish was reached (i.e., after 7-days of culturing). The culture fluid was composed of (with a 2:1 ratio) Neurobasal (NB) medium (Gibco), supplemented with B-27, 2 mM L-glutamine, 10 μg/ml penicillin/streptomycin and 25 μM β-mercaptoethanol, and Minimal Essential medium (MEM). All materials were purchased from Thermo Fischer (Life Technologies). Note that, 5'-deoxy-5-fluorouridine (5'-DFUR) was also added to the medium 24 h after cell seeding to kill unwanted proliferating cells (such as fibroblasts and glial cells) while normal neurons remained largely unaffected.

### Indirect Immunofluorescence of NCAM

**Figure 1:**
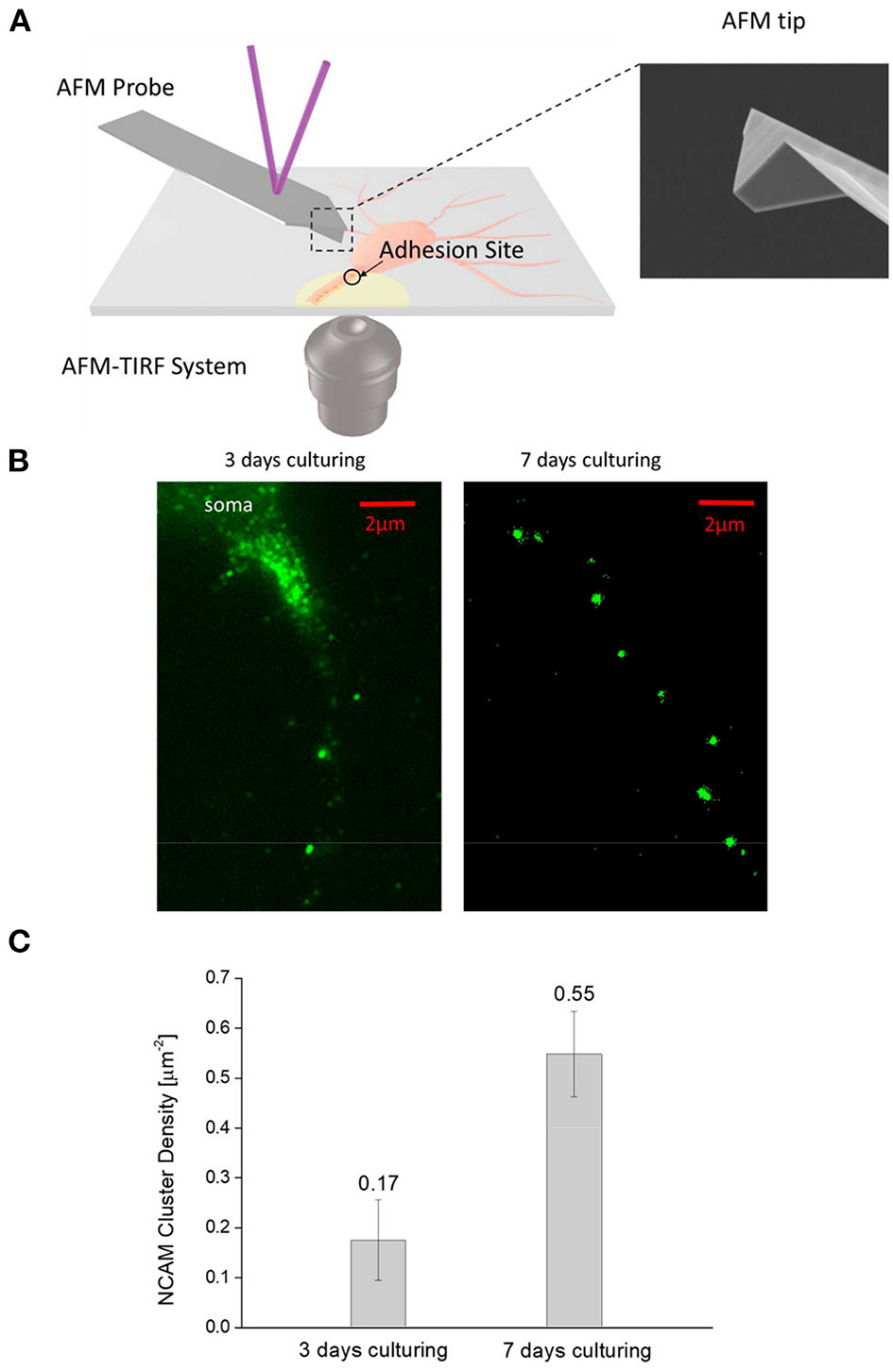
**(A)**—Schematic diagram of the combined AFM—TIRFM system for monitoring the adhesion and injury response of neural cells. **(B)**—Typical NCAM fluorescent image of 3-days and 7-days culturing neurons taken by the TIRFM. Interestingly, NCAMs tend to aggregate into small clusters (bright spots) at the axon-substrate interface in both figures. However, denser cluster is observed in 7-day neurons compared to 3-day ones. **(C)**—Areal density of NCAM clusters (calculated by dividing the total number of NCAM clusters observed in the TIRFM image by the apparent axon-substrate contact area) in 3-day neurons is significantly lower (α = 0.01, *n* = 10) lower than that in 7-day ones.

Cultured neurons were first rinsed with balanced salt solution (BSS) and incubated at room temperature and pH 7.4 for 7 min with 4% paraformaldehyde in BSS. After that, cells were loaded with monoclonal anti-NCAM antibody (Sigma) (for 1 h) and then incubated for another 1 h at room temperature with secondary anti-mouse IgG Alexa Fluor 488 (Invitrogen). The adhesion patterns formed at the axon-substrate interface were monitored by a TIRFM placed underneath the coverslip (Figure 1A).

### AFM-Based Transection of Axons

Axon transection was achieved by a sharp AFM (JPK Instruments, NanoWizard II) probe fabricated by focused ion beam milling (refer to the inset of Figure 1A). Specifically, after approaching the tip to the cell gradually, a compressive force (60 nN) was applied onto the axon. At the same time, a quick lateral slice (by the tip) was conducted through manual manipulation to complete the transection. The triggered response of axon was then recorded for 30 min and analyzed. In particular, the retraction distance of axon was quantified from time lapse images with NeuronGrowth, an ImageJ plugin software developed by Fanti et al. (2008), while subsequent statistical analysis was performed with the software OriginPro.

## Results

### Maturation of Neurons Increases the Density of Adhesion Clusters at the Axon-Substrate Interface

We first examined how the maturation process of neural cells affects their adhesion with the outside. Typical TIRFM images of the stained NCAMs at the axon-substrate interface of 3-day and 7-day cultured neurons are given in Figure 1B which clearly indicates that these molecules tend to aggregate into small clusters. Given that NCAM is a unique carrier of the polyanionic carbohydrate, polysialic acid (PSA) (Kiselyov et al., 2005; Dityatev and El-Husseini, 2006), allowing them to physically bind to the positively charged PLL on the coverslip, it is likely that strong adhesions are formed in these locations (Liu et al., 2020). Interestingly, although the size of NCAM clusters (~0.5–1 μm) was found to be more or less the same in mature (7-day) and immature (3-day) neurons, the areal density of such clusters was three times higher in mature axons (Figure 1C) indicating that a much stronger adhesion with outside will be established as neuron matures.

### Immature Neurons Exhibit Larger Retraction Response to Injury

**Figure 2:**
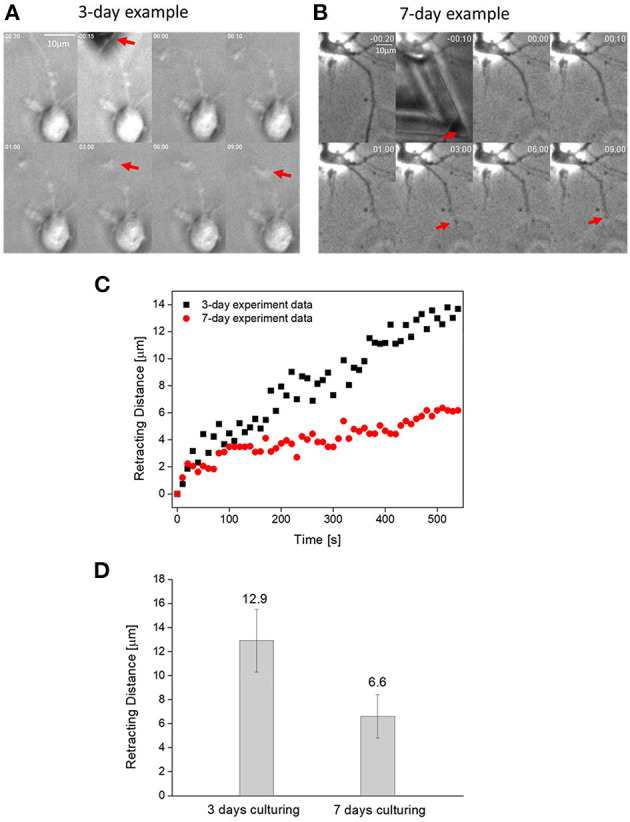
**(A)** Time lapse images showing the transection-induced retraction of a 3-day axon. Here, position of the transected end of axon is indicated by the red arrow. **(B)** Time lapse images showing the transection-induced retraction of a 7-day axon. **(C)** Representative retraction trajectories of a mature (7-days of culturing) and immature (3-days of culturing) neuron. **(D)** Comparison between the average retraction distance of 3- and 7-day axons (α = 0.01, *n* = 15).

Next, we used a sharp AFM probe to transect axons on 3- and 7-day neurons and then monitored their retraction response. Representative time lapse images of such test are shown in Figures 2A,B with the corresponding retraction trajectories of the transected end of axon given in Figure 2C. Since previous studies have shown that the injury-induced retraction distance of an axon is influenced by its so-called floating length (where axon-substrate adhesion was totally disrupted during the transection process, refer to Shao et al. (2019) and the distance between the transection site to soma, we have intentionally transected axons ~50 μm away from the main cell body as well as only analyzed cases where the floating length was around/ <15 μm. Interestingly, as shown in Figures 2C,D, the retraction distance of immature (3-day) axons was found to be more than twice of that for mature (7-day) ones. In addition, the retraction curves of 3-day axons were decorated with sudden bursts, presumably due to the disruption of individual adhesion clusters.

### A Physical Model for Explaining the Maturation-Dependent Retraction Response of Neurons

**Figure 3:**
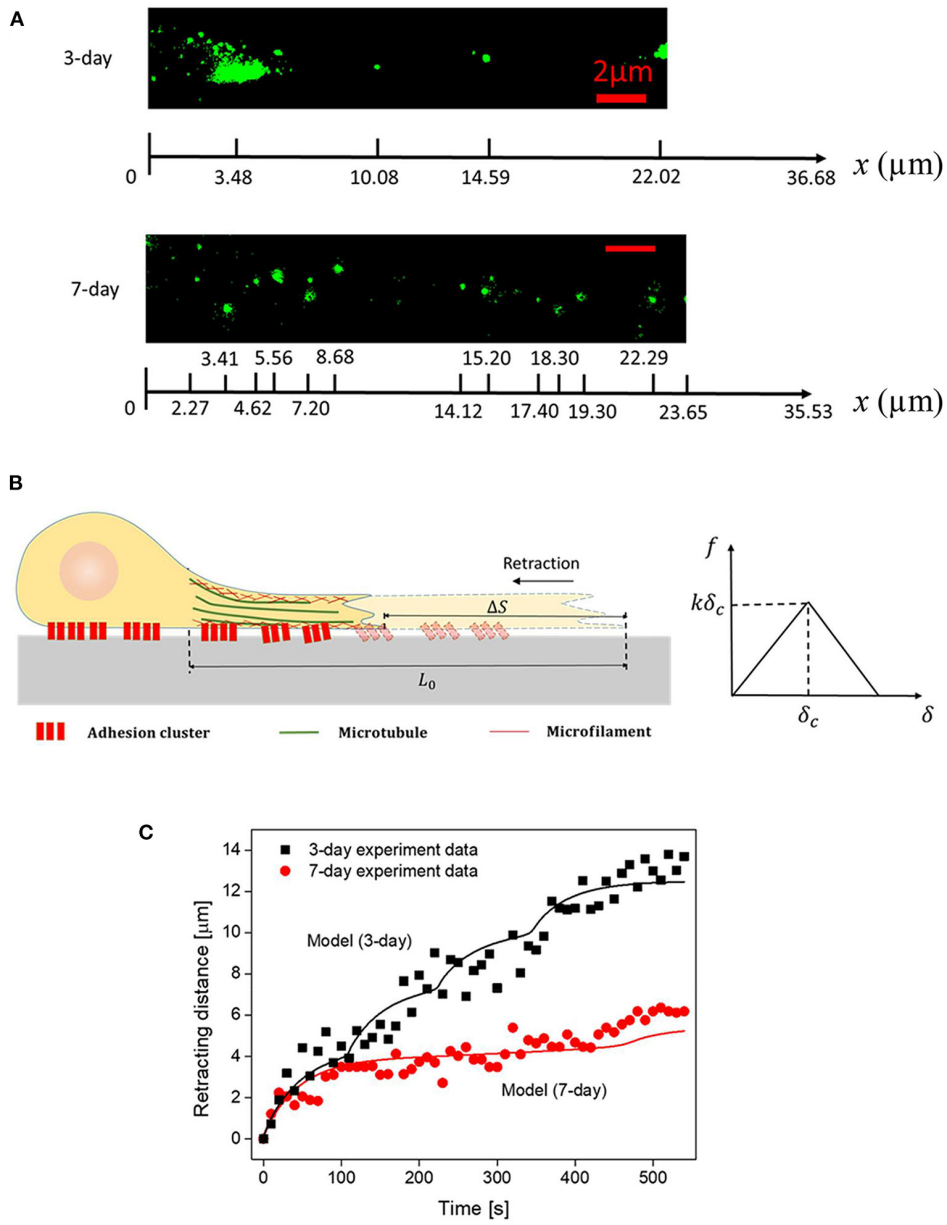
**(A)** Representative spatial distributions of NACM clusters (in 3- and 7-day axons) captured by TIRFM. **(B)** Schematics of the axon structure and cohesive law used in the model. **(C)** Comparison between the recorded and simulated retraction trajectories of axons. Parameters adopted in the simulation are summarized in Table 1.

It is conceivable that the reduced retraction response of mature neurons to injury is caused by the enhanced adhesion formed between the axon and outside. Following this line of reasoning, we tried to explain our experimental observations with a physical developed recently (Shao et al., 2019). Specifically, we assumed that the observed retraction is driven by axonal tension and resisted by cell-substrate adhesion (see Figure 3A). Since the whole retraction process occurs relatively slowly (i.e., taking minutes to complete), the axon can be assumed to be in quasi-static equilibrium, that is the axonal tension must be balanced by cell-substrate adhesion at any given moment. In this case, a small segment of axon (with length dx) will be subject to internal forces acting on the two ends, along with the adhesion force at the axon-substrate interface (Figure 3B). Balance of forces requires

(1) $$\begin{array}{r} A\frac{\partial \sigma \left(x,t\right)}{\partial x}+w\cdot f\left(x,t\right)=0, \end{array}$$

where σ, *A* and *w* are the internal stress, cross-section area and width of the axon, respectively. *f* represents the adhesion force density exerted on the cell. For simplicity, the axon was treated as a viscoelastic Kelvin–Voigt solid (taking into account both its elastic and viscous responses) with the internal stress given by

(2) $$\begin{array}{r} \sigma =E\frac{\partial u}{\partial x}+\eta \frac{\partial^{2}u}{\partial x\partial t} \end{array}$$

with *u*, *E* and η representing the displacement, apparent elastic modulus and viscosity of axon. Next, a cohesive law was introduced to connect adhesion force with the spatial distribution of NCAM molecules as well as the behavior of NCAM-PLL bonds responsible for bringing two surfaces together. Specifically, here *f* was assumed to take the following form

(3) $$\begin{array}{r} f\left(x,t\right)=\rho \left(x\right)\cdot k\delta \left(1-D_{n}\right), \end{array}$$

where ρ(*x*) represents the areal density distribution of NCAMs along the axon (which can be estimated from the TIRFM images obtained), δ and *k* stand for the stretching (due to the relative sliding between the axon and substrate) and stiffness of the NCAM-PLL bond. The enforced disruption of adhesion was captured by *D*_*n*_ defined as

(4) $$D_{n}=\{\begin{array}{cc} 0 & \delta \leq \delta_{c}\\ \frac{2(\delta -\delta_{c})}{\delta } & \delta_{c}<\delta <2\\ 1 & \delta \geq 2\delta_{c} \end{array}\delta_{c}.$$

Essentially, δ = u(x, 0)−u(x, t) is the magnitude of the relative sliding between the cell and the substrate, and δ_*c*_ represents the critical distance where deformed NCAM-PLL bonds start to break. Consistent with adhesions in biological systems, Equation (4) means that the bond force increases linearly with δ initially until it reaches the threshold value δ_*c*_, beyond which the force begins to decrease with δ before rupture of the bond taking place at δ = 2δ_*c*_ (where total debonding occurs, i.e., f = 0 beyond this critical sliding distance). It should be noted that ρ(*x*) comes from the fluorescent (NCAM staining) figures captured by TIRFM, for example Figure 3A. The length of the cluster was assumed to be 1 um and NCAM-PLL bond was assumed to be equally distributed in each cluster with a spacing 38 nm for simplicity.

By choosing realistic parameters E = 5 kPa, τ = E/η = 80s, *w* = 1 μm, ε*Ew*^2^ = 2.5 nN (pre-existed tension within the axon), *k* = 0.4 pN/nm, *kδ*_*c*_ = 104 pN (refer to Table 1), the retraction curves of 3- and 7-day neurons were simulated (Figure 3C). Note that, the observed TIRFM images of NCAM clusters (Figure 3A) were used as input for the adhesion distribution function ρ(*x*). Essentially, the length of each cluster was taken to be 1 μm while a constant spacing *d* of 38 nm between NCAM-PLL bonds was assumed within the cluster (i.e., ρ(*x*) equals to *1/d* within the spatially distributed 1 μm long clusters and 0 everywhere else). Interestingly, the recorded retraction curves (decorated with sudden bursts) for both 3- and 5-day neurons were well-explained by the model (Figure 3C), indicating the essential features involved in the inhomogeneous adhesion-regulated retraction of axon have been captured by this description. Finally, given that the average densities of adhesion clusters for 3- and 7-day neurons were measured as 0.17 and 0.55 μ*m*^−2^, the apparent adhesion energy γ for immature or mature neural cells was estimated to be ~0.12 and 0.39 mJ/m^2^, respectively.

**Table 1:** List of parameters in the model.

	**Parameter meaning**	**Adopted values**	**References**
E	Elastic modulus of axon	5 kPa	4.6 ± 1.5 kPa (Grevesse et al., 2015)
τ	Characteristic relaxation time of axon	80 s	50–2,000 s (Bernal et al., 2007)
*w*	Width of the axon	1 μm	Directly measured
ε*Ew*^2^	Pre-existed tension inside axon	2.5 nN	~2 nN (Mutalik et al., 2018)
k	Stiffness of the adhesion bond	0.4 pN/nm	~1 pN/nm (Fisher et al., 1999; Wieland et al., 2005)
kδc	Maximal force an adhesion bond can sustain for 3-days	104 pN	Tens to hundreds of pico-newton (Wieland et al., 2005)
d	NCAM-PLL bond spacing	38 nm	~28–73 nm (Arnold et al., 2004; Jiang et al., 2015)
ρ_0_	Areal density of adhesion clusters for 3-day neurons	0.17 μm^−2^	Directly measured
	Areal density of adhesion clusters for 7-day neurons	0.55 μm^−2^	Directly measured
γ	Apparent adhesion energy density for 3-day neurons	0.12 mJ/m^2^	0.1–0.4 mJ/m^2^ (Liu et al., 2020)
	Apparent adhesion energy density for 7-day neurons	0.39 mJ/m^2^	

## Discussion

In this brief report, we showed how maturation of neural cells affects their adhesion with outside as well as their response against traumatic injury. Specifically, it was found that ~3 times more NCAM-mediated adhesion clusters were formed at the interface of matured axons (7-day of culturing) and substrate, compared to immature (3-day of culturing) ones, although their size (~1 μm) remained more or less the same in both cases. Interestingly, the weaker adhesion in immature axons led to a stronger retracting response against injury, along with a retraction curve decorated with sudden bursts. A physical model was also adopted to explain the observed retraction trajectories which suggested that the observed displacement excursions are due to the disruption of individual adhesion clusters and the apparent axon-substrate adhesion energy increases from ~0.12 to 0.39 mJ/m^2^ as neural cell matures, in agreement with recent experiments (Liu et al., 2020). Given the critical role of axon adhesion/retraction in the formation/disintegration of the neural network, findings here can enhance our basic understanding of brain injury. In addition, the fact that the injury-response of neurons was regulated by their interactions with outside could provide clues for the development of neural engineering.

One thing must be pointed out is that maturation was assumed to only influence neuron-substrate adhesion in the present model. In reality, it has been reported that a much more organized actin skeleton (Zhong et al., 2014) will be developed in the mature axons, compared to immature ones, indicating that the bulk mechanical response (Lin, 2009) of axon could change considerably as neuron matures. In addition, evidence also indicated that NCAM clusters can be connected to the cell cortex through spectrin binding (Leshchyns'ka et al., 2003), suggesting that cortical actomyosin contraction may play a role in the formation/disruption of mature adhesions and how they respond to external stimuli as well. Evidently, carefully designed studies are needed in the future to address these important issues.

## Data Availability Statement

The raw data supporting the conclusions of this article will be made available by the authors, without undue reservation.

## Author Contributions

YL and ZC conceived the study. XS and MS performed the experiments. XS, CF, RC, and YL analyzed the data. XS, CF, ZC, and YL developed the physical model. XS, ZC, and YL wrote the manuscript. All authors reviewed the manuscript. All authors contributed to the article and approved the submitted version. All authors contributed to the article and approved the submitted version.

## Conflict of Interest

The authors declare that the research was conducted in the absence of any commercial or financial relationships that could be construed as a potential conflict of interest.
